# An evolutionarily conserved function of C-reactive protein is to prevent the formation of amyloid fibrils

**DOI:** 10.3389/fimmu.2024.1466865

**Published:** 2024-09-16

**Authors:** Alok Agrawal, Asmita Pathak, Donald N. Ngwa, Avinash Thirumalai, Peter B. Armstrong, Sanjay K. Singh

**Affiliations:** ^1^ Department of Biomedical Sciences, Quillen College of Medicine, East Tennessee State University, Johnson City, TN, United States; ^2^ Marine Biological Laboratory, Woods Hole, MA, United States

**Keywords:** C-reactive protein, *Limulus polyphemus*, American horseshoe crab, amyloidosis, protein toxicity

## Abstract

C-reactive protein (CRP) binds to phosphocholine (PCh)-containing substances and subsequently activates the complement system to eliminate the ligand. The PCh-binding function of CRP has been conserved throughout evolution from arthropods to humans. Human CRP, in its structurally altered conformation at acidic pH, also binds to amyloid-β (Aβ) and prevents the formation of Aβ fibrils. It is unknown whether the Aβ-binding function of CRP has also been evolutionarily conserved. The aim of this study was to determine whether CRP isolated from American horseshoe crab *Limulus polyphemus* was also anti-amyloidogenic and whether this function required structural alteration of *Limulus* CRP (Li-CRP). Two CRP species Li-CRP-I and Li-CRP-II were purified from hemolymph by employing PCh-affinity chromatography and phosphoethanolamine-affinity chromatography, respectively. Both Li-CRP-I and Li-CRP-II bound to immobilized Aβ at physiological pH. Unlike human CRP, Li-CRP did not require any changes in its overall structure to bind to Aβ. Both Li-CRP-I and Li-CRP-II bound to Aβ in the fluid phase also and prevented the fibrillation of Aβ. Additionally, ion-exchange chromatography of purified Li-CRP indicated that a variety of Li-CRP molecules of different subunit compositions were present in *Limulus* hemolymph, raising the possibility that the presence of various Li-CRP species in hemolymph facilitates the recognition of a range of proteins with differing amyloidogenicity. We conclude that the binding of CRP to Aβ is an ancient function of CRP. In invertebrates, the Aβ-binding function of CRP can protect the host from toxicity caused by amyloidogenic and pathogenic proteins. In humans, the Aβ-binding function of CRP can protect against inflammatory diseases in which the host proteins are ectopically deposited on either host cells or foreign cells in an inflammatory milieu since immobilized proteins may expose Aβ-like structures after deposition at places where they are not supposed to be.

## Introduction

C-reactive protein (CRP) is characterized by its ability to bind to phosphocholine (PCh) or to both PCh and phosphoethanolamine (PEt) in a Ca^2+^-dependent manner (1–6). Human CRP is encoded by a single gene producing a non-glycosylated polypeptide of 206 amino acid residues and is a cyclic pentamer of five identical non-covalently attached subunits (7). The molecular weights of the pentamer and subunits are ~115 kDa and ~23 kDa, respectively. The interfaces of the CRP pentamer are flexible (8–10). Human CRP also has the ability to multimerize as a stack of two pentamers in the presence of zinc ions or high levels of NaCl (10–12).

Human CRP functions in two different conformations: as a native pentamer at physiological pH and as a non-native pentamer at acidic pH (13). When exposed to acidic pH, the native pentameric conformation of CRP is altered (8, 10, 14–17). Such non-native pentameric CRP is also generated by exposure of native CRP to H_2_O_2_ (16, 18). Native CRP binds primarily to PCh-containing substances and subsequently activates the complement system to eliminate the ligand (19, 20). Non-native CRP binds to amyloid-β peptide 1-42 (Aβ) and to Aβ-like structures displayed on immobilized, denatured and aggregated proteins, in addition to retaining the ability to bind to PCh (15, 21–24). It has also been recently shown that non-native pentameric CRP binds to Aβ in the fluid phase, subsequently preventing their fibrillation (21). Thus, human CRP is an anti-amyloidogenic protein; however, an inflammatory milieu and subsequent conformational changes in native CRP are required for human CRP to be anti-amyloidogenic.

CRP has been conserved throughout evolution (25–27). The earliest known CRP is reported from arthropods American horseshoe crab *Limulus polyphemus* (28), Japanese horseshoe crab *Tachypleus tridentatus* (29) and South Asian horseshoe crab *Carcinoscorpius rotundicauda* (30). CRP has also been isolated and characterized from the mollusk *Achatina fulica* (31, 32). It is unknown whether the anti-amyloidogenic function of human CRP has been evolutionarily conserved and whether CRP from invertebrates exhibits structure-based ligand-binding properties.

CRP from *L. polyphemus* which diverged from the vertebrate lineage ~500 million years ago (35) has been previously investigated in greater detail (28, 33–38): *Limulus* CRP (Li-CRP) is encoded by three homologous genes producing three types of subunits (34). All three subunit types have 218 amino acid residues and are present approximately in equimolar amounts (35). They also share an identical N-terminal sequence of 44 amino acid residues and an identical C-terminal sequence of 13 amino acid residues (35). There is only 10% microheterogeneity amongst the rest of the amino acid sequences. There are six half-cystines that form the three intrachain disulfide bonds in all subunits (35). The positions of six half-cystines are constant in all subunits. All three types of subunits are glycosylated although the glycosylation is variable (35). The sites of glycosylation are also constant in all three subunits. Each type of subunit forms a hexagonal structure as revealed by electron microscopy and two hexamers are stacked together to form the Li-CRP dodecamer. The molecular weights of Li-CRP dodecamer and subunits are ~300 kDa and ~25 kDa, respectively (36). Thus, there are twelve subunits in each Li-CRP molecule. Each type of subunit binds to both PCh and PEt in a Ca^2+^-dependent manner (34). There is no immunological cross-reactivity between Li-CRP and human CRP (28, 39).

There are two other proteins similar to Li-CRP present in *Limulus* hemolymph. One has been named human serum amyloid P component (SAP)-like protein which has an affinity for PEt and carbohydrate moieties but not PCh (33, 40–42). The N-terminal amino acid sequences of Li-CRP and SAP-like protein are different. The first ten N-terminal amino acid residues of Li-CRP are LEEGEITSKV/I and of SAP-like protein are AVDIRDVKIS (34, 35, 41). The other protein similar to Li-CRP in hemolymph has been named limulin which binds to fetuin or sialic acid (28, 33, 43–45).

The aim of this study was to determine whether Li-CRP was an anti-amyloidogenic protein like human CRP and whether this function of Li-CRP required acidic pH. Since Li-CRP is known to bind to both PCh and PEt, we isolated Li-CRP from the hemolymph employing two different affinity chromatography methods. Li-CRP isolated by PCh-affinity chromatography was named Li-CRP-I and Li*-*CRP isolated by PEt-affinity chromatography was named Li-CRP-II. Both CRP species, Li-CRP-I and Li-CRP-II, were then characterized and investigated for their effects on Aβ fibrillation.

## Materials and methods

### Purification of human CRP

Human CRP was purified from discarded body fluids employing PCh-affinity chromatography, exactly as described previously (46).

### Purification of Li-CRP-I and Li-CRP-II

*Limulus* hemolymph was obtained from Marine Biological Laboratory. Li-CRP was purified from the hemolymph as described previously, with some modifications (33). Hemolymph was first centrifuged at 15,000 g for 15 min to collect the clear blue plasma. The plasma was passed through a Sepharose 4B (MilliporeSigma, 4B200) column in 10 mM Tris-HCl, pH 7.2, containing 150 mM NaCl (TBS) and 2 mM CaCl_2_ to remove carbohydrate-binding proteins. Next, polyethylene glycol-8000 was added to the plasma at a final concentration of 3% and incubated at 4°C for 16 h with shaking to remove hemocyanin. The plasma was then centrifuged at 30,000 g for 30 min. The blue pellet was discarded, and the transparent plasma was recovered and used to purify Li-CRP by employing two different affinity chromatography methods, as follows.

Method 1: Li-CRP was isolated from plasma by employing Ca^2+^-dependent PCh-affinity chromatography as described previously for purification of human CRP (46). Briefly, plasma was passed through a PCh-Sepharose column in a Ca^2+^-containing buffer. After washing the column, Li-CRP was eluted by using EDTA. The EDTA eluate was concentrated and Li-CRP was further purified by using gel filtration chromatography on a Superose 12 column in TBS containing 5 mM EDTA. Fractions containing Li-CRP were pooled and dialyzed against TBS containing 2 mM CaCl_2_. This preparation of pure Li-CRP which involved purification by PCh-affinity chromatography was named Li-CRP-I.

Method 2: Li-CRP was isolated from plasma by employing Ca^2+^-dependent PEt-affinity chromatography as described previously for purification of recombinant human CRP mutants (46). Briefly, plasma was passed through a PEt-Sepharose column in a Ca^2+^-containing buffer. After washing the column, Li-CRP was eluted by EDTA. The EDTA eluate was concentrated and Li-CRP was further purified as described above in method 1. This preparation of pure Li-CRP which involved purification by PEt-affinity chromatography was named Li-CRP-II.

The purity of Li-CRP preparations was determined by using denaturing SDS-PAGE under reducing conditions in a 4-20% gradient gel and by N-terminal sequencing. The N-terminal amino acid sequencing of Li-CRP was performed at the Molecular Structure Facility, University of California, Davis. The concentrations of Li-CRP-I and Li-CRP-II were determined by using the extinction coefficient of 15.49 at A_280_ (36).

### Determination of the overall structure of Li-CRP

The molecular weight of native Li-CRP was determined by employing gel filtration chromatography on a calibrated Superose 12 column (10/300 GL, GE healthcare), as described previously for human CRP (46). The gel filtration column was equilibrated with TBS containing 5 mM EDTA. Li-CRP was injected into the column and eluted with TBS containing 5 mM EDTA at a flow rate of 0.3 ml/min. Fractions (60 fractions, 250 μl each) were collected and absorbance at 280 nm measured to locate the elution volume of Li-CRP. The molecular weight of the subunits of Li-CRP was determined by employing denaturing SDS-PAGE under reducing conditions in a 4-20% gradient gel. The composition of Li-CRP was further evaluated by employing anion exchange chromatography on a MonoQ column (5/50 GL, GE healthcare), as described previously for human CRP (46). The fractions collected after the chromatography were subjected to denaturing SDS-PAGE under reducing conditions in a 4-20% gradient gel.

### Deglycosylation of Li-CRP

Deglycosylation of Li-CRP was performed using the Glycoprofile IV Chemical Deglycosylation kit (MilliporeSigma, PP0510) according to manufacturer’s instructions. Briefly, 150 µl of chilled trifluoromethanesulfonic acid (MilliporeSigma, 34781-7) was added to 1 mg of cold lyophilized Li-CRP and incubated on ice for 25 min with occasional shaking. Bromophenol blue (0.2%; 4 µl) was added to the reaction followed by dropwise addition of 60% pyridine solution (MilliporeSigma, P5496) in a methanol-dry ice bath until the color of the reaction changed from red to light purple or blue. Samples were then dialyzed against TBS overnight. The deglycosylation of Li-CRP was verified and the molecular weight of the deglycosylated subunits determined by employing denaturing SDS-PAGE under reducing conditions in a 4-20% gradient gel.

### Generation of polyclonal anti-Li-CRP-I antibodies

Li-CRP-I was purified as described above. Rabbit polyclonal antibodies against purified Li-CRP-I were generated commercially by Thermo Fisher Scientific. The antiserum obtained from the company was used as such after titrating to determine the dilution to be used in the assays.

### Immunological cross-reactivity assay

The immunological cross-reactivity between Li-CRP-I and Li-CRP-II was determined by employing anti-Li-CRP-I antibodies and purified Li-CRP-I and Li-CRP-II, as follows. Microtiter wells were coated with increasing amounts of Li-CRP-I or Li-CRP-II and incubated at 37°C for 2 h. The unreacted sites in the wells were blocked with TBS containing 0.5% gelatin for 45 min at room temperature. Next, anti-Li-CRP-I antibodies diluted in TBS was added to the wells and incubated at 37°C for 1 h. HRP-conjugated donkey anti-rabbit IgG, diluted in TBS, was used as the secondary antibody. Color was developed using ABTS as the substrate and the OD was read at 405 nm in a microtiter plate reader.

### PCh-binding assay

Binding activity of Li-CRP for PCh was measured by using pneumococcal C-polysaccharide (PnC; purchased from Statens Serum Institut) as the PCh-containing ligand, exactly as described previously (9).

### Preparation of Aβ peptides, monomers and fibrils

Lyophilized Aβ peptide 1-42 was purchased from Bachem (H-1368) and reconstituted according to manufacturer’s instructions. Reconstituted Aβ peptide solution should contain Aβ monomers; however, oligomers of Aβ monomers are also present in the reconstituted Aβ peptide solution (47). Aβ monomers were prepared from Aβ peptides according to a published method (48) and exactly as described previously (21). In brief, lyophilized Aβ peptides were dissolved in hexafluoroisopropanol (MilliporeSigma) and incubated at 37°C for 2 h for monomerization of the oligomers present in the Aβ peptide solution. After removing hexafluoroisopropanol by evaporation overnight, the vials containing the film of Aβ monomers were stored at -20°C. Aβ fibrils were prepared from Aβ peptides according to a published method (48) and exactly as described previously (21). In brief, when needed, ice-cold TBS was added to a vial containing the film of Aβ monomers to obtain a 200 μg/ml solution of Aβ monomers. To prepare Aβ fibrils, the Aβ monomers were vortexed and transferred to a 96-well microtiter plate (Immunochemistry Technologies, Costar 266). The plate was incubated at 37°C for 3 h with shaking at 300 rpm. The resulting Aβ fibrils were stored at -20°C until needed.

### Li-CRP-Aβ binding assay

Binding activity of Li-CRP for immobilized Aβ was evaluated as described earlier (21). Briefly, lyophilized Aβ peptides were reconstituted in TBS. Microtiter wells were coated with 10 μg/ml of Aβ at 4°C overnight. The unreacted sites in the wells were blocked with TBS containing 0.5% gelatin. Li-CRP, diluted in TBS containing 0.1% gelatin, 0.02% Tween-20 and 2 mM CaCl_2_ (TBS-Ca), was added in duplicate wells and incubated at 37°C overnight. After washing the wells, rabbit polyclonal anti-Li-CRP-I antibody was used to detect bound Li-CRP. HRP-conjugated donkey anti-rabbit IgG (GE Healthcare) was used as the secondary antibody. Color was developed, and the OD was read at 405 nm.

### Human SAP-Aβ binding assay

Binding activity of human SAP (Calbiochem, 565190) for immobilized Aβ was evaluated as follows. Microtiter wells were coated with 10 µg/ml of Aβ peptide, monomer and fibrils diluted in TBS. The unreacted sites in the wells were blocked with TBS containing 0.5% gelatin for 45 min at room temperature. SAP, diluted in TBS-Ca was then added in duplicate wells and incubated for 2 h at 37°C. Bound SAP was detected by using rabbit anti-human SAP (Calbiochem, 565191) by adding the antibody (5 µg/ml) to the wells and incubating at 37°C for 1 h. HRP- conjugated donkey anti-rabbit antibody (Southern Biotech, 6441-05) was used as the secondary antibody. Color was developed, and the OD was read at 405 nm.

### Thioflavin T assay for Aβ fibrillation

The fibrillation of Aβ monomers was monitored by employing ThT assay according to a published method (49, 50) and exactly as described previously (21). In brief, the effects of Li-CRP on Aβ fibrillation were determined by adding CRP to Aβ at time zero, that is, CRP and Aβ were mixed together before the beginning of the fibrillation reaction. The fibrillation reaction mix was prepared with and without CRP. In CRP-containing mixture, the final concentrations of CRP were 1, 10 and 100 μg/ml. After vortexing, 240 μl of each mixture was transferred in triplicate wells in a 96-well microtiter plate (Immunochemistry Technologies, Costar 266). Fluorescence was measured by using the Synergy H1 microplate reader (BioTek) with excitation at 440 nm and emission at 480 nm. After the first measurement at 5 min, the plate was incubated at 37°C for 3 h with shaking at 300 rpm; fluorescence was measured every 15 min for 3 h.

## Results

### Verification of the composition and characteristics of Li-CRP-I and Li-CRP-II

**Figure 1:**
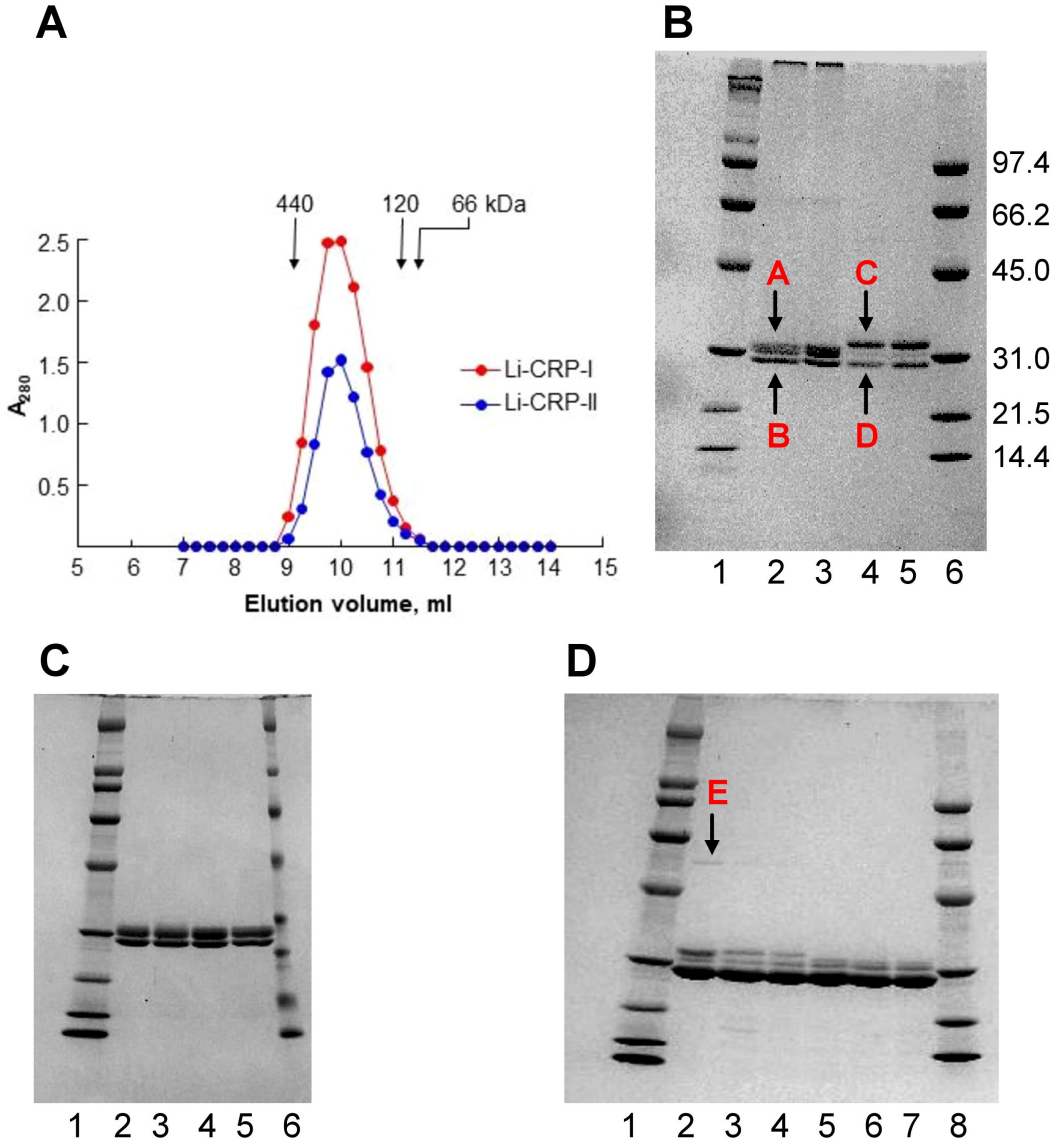
Purification of Li-CRP-I and Li-CRP-II. **(A)** Elution profiles of Li-CRP-I and Li-CRP-II from the gel filtration column are shown. The arrows point to the elution volumes of molecular weight markers: apoferritin (440 kDa), human CRP (120 kDa) and BSA (66 kDa). A representative of three chromatograms is shown. **(B)** Li-CRP-I and Li-CRP-II were subjected to SDS-PAGE. Lane 1, Bio-Rad broad-range molecular weight markers; Lane 2, Li-CRP-I eluted from the PCh-affinity column by using EDTA; Lane 3, Li-CRP-I eluted from the PCh-affinity column by using PCh; Lane 4, Li-CRP-II eluted from the PEt-affinity column by using EDTA; Lane 5, Li-CRP-II eluted from the PEt-affinity column by using PEt; Lane 6, Bio-Rad low-range molecular weight markers. The molecular weights of the CRP subunits were calculated from three separate gels and presented as average ± SEM (see the Results section). **(C)** Lane 1, molecular weight markers; Lane 2, Li-CRP-I eluted from the PCh-affinity column by using EDTA; Lane 3, Li-CRP-I eluted from the PCh-affinity column by using EDTA, followed by gel filtration chromatography; Lane 4, Li-CRP-I eluted from the PCh-affinity column by using PCh; Lane 5, Li-CRP-I eluted from the PCh-affinity column by using PCh, followed by gel filtration chromatography; Lane 6, molecular weight markers. **(D)** Lane 1, molecular weight marker; Lane 2, Li-CRP-II eluted from the PEt-affinity column by using EDTA; Lane 3, Li-CRP-II eluted from the PEt-column by using EDTA, followed by ion-exchange chromatography; Lane 4, Li-CRP-II eluted from the PEt-column by using EDTA, followed by gel filtration chromatography; Lane 5, Li-CRP-II eluted from the PEt-affinity column by using PEt; Lane 6, Li-CRP-II eluted from the PEt-column by using PEt, followed by ion-exchange chromatography; Lane 7, Li-CRP-II eluted from the PEt-column by using PEt, followed by gel filtration chromatography; Lane 8, molecular weight markers. A representative of three Coomassie brilliant blue-stained gels is shown for each panel.

Gel filtration and SDS-PAGE were performed to determine the molecular weight of native Li-CRP, the number of subunits in each molecule and the molecular weight of the subunits. As shown in **Figure 1A**, the elution volumes from the calibrated gel filtration column for both Li-CRP-I purified by PCh-affinity chromatography and Li-CRP-II purified by PEt-affinity chromatography were 10 ml. The molecular weights of both Li-CRP-I and Li-CRP-II were calculated to be ~300 kDa. SDS-PAGE (**Figure 1B**) revealed that Li-CRP-I was composed of two types of subunits of molecular weight 27.3 ± 1.7 kDa (band A) and 25.8 ± 2.7 kDa (band B). The band A was a doublet of two bands. Li-CRP-II was also composed of two types of subunits of molecular weight 29.6 ± 2.6 kDa (band C) and 24.7 ± 2.5 kDa (band D), although a third subunit of molecular weight 28.2 ± 2.5 kDa was also seen as a faint band in between bands C and D. In another preparation of Li-CRP-II, there was an additional faint band of molecular weight 54.8 ± 0.01 (band E, **Figure 1D**). Thus, both Li-CRP-I and Li-CRP-II were composed of twelve subunits, assuming an average molecular weight of 25 kDa for each subunit.

We checked the purity of Li-CRP-I eluted by using either EDTA (lane 2) or PCh (lane 3) from the PCh-affinity column (**Figure 1C**). We also compared the purity of Li-CRP-I purified by either affinity chromatography (lanes 2-3) only or by affinity chromatography followed by gel filtration (lanes 4-5). As shown, there was no difference in the purity of Li-CRP-I eluted by either EDTA or PCh from the affinity column. Similarly, there was no difference in the purity of Li-CRP-I purified by just affinity chromatography or by affinity chromatography followed by gel filtration. These results indicated that the affinity-purified Li-CRP-I was pure; there was no need for further purification.

The purity of Li-CRP-II eluted by using either EDTA (lanes 2-4) or PEt (lanes 5-7) from the PEt-affinity column was also investigated (**Figure 1D**). As shown, when Li-CRP-II was eluted from the affinity column by using PEt (lane 5), the top band C (as shown in **Figure 1B**) was absent; instead, there was a doublet of the middle band. We also compared the purity of Li-CRP-II purified by either affinity chromatography (lanes 2 and 5) alone or by affinity chromatography followed by ion-exchange chromatography (lanes 3 and 6) or by affinity chromatography followed by gel filtration (lanes 4 and 7). There was no difference in the purity of Li-CRP-II purified by just affinity chromatography or by affinity chromatography followed by either ion-exchange chromatography or gel filtration. These results indicated that the affinity-purified Li-CRP-II was also pure; there was no need for further purification.

**Figure 2:**
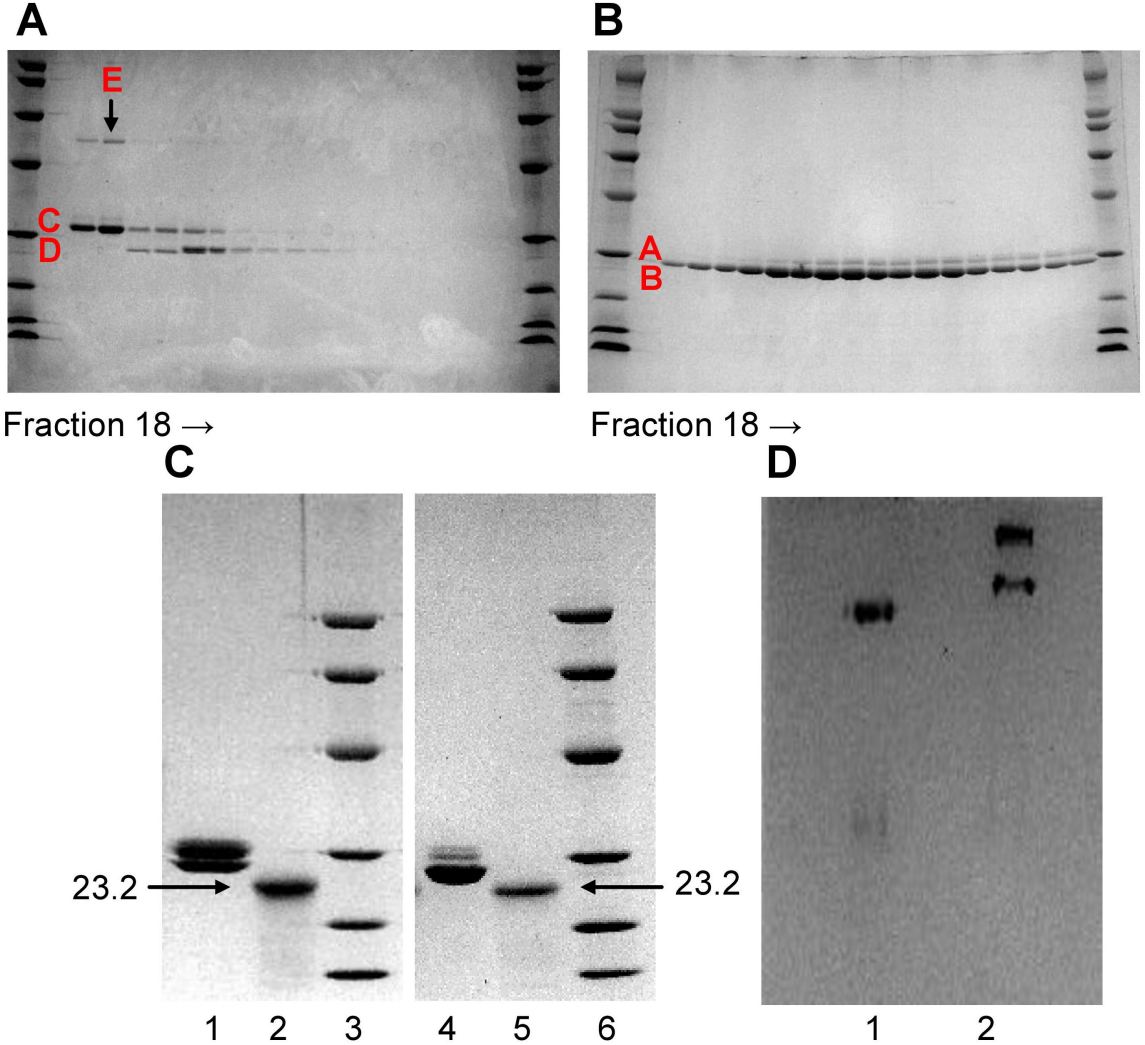
Characterization of Li-CRP-I and Li-CRP-II. **(A)** Li-CRP-II, purified by affinity chromatography and gel filtration, was subjected to ion-exchange chromatography on a MonoQ column (chromatogram not shown). Fractions (18-35) collected from the MonoQ column were subjected to SDS-PAGE. **(B)** Li-CRP-I, purified by affinity chromatography and gel filtration, was subjected to ion-exchange chromatography on a MonoQ column (chromatogram not shown). Fractions (18-35) collected from the MonoQ column were subjected to SDS-PAGE. **(C)** Native and deglycosylated Li-CRP-I and Li-CRP-II were subjected to SDS-PAGE. Lane 1, Li-CRP-I (10 μg); Lane 2, deglycosylated Li-CRP-I (80 μg); Lane 3, molecular weight markers; Lane 4, Li-CRP-II (10 μg); Lane 5, deglycosylated Li-CRP-II; Lane 6, molecular weight markers. **(D)** Li-CRP-I and Li-CRP-II were subjected to native PAGE. Lane 1, Li-CRP-I; Lane 2, Li-CRP-II. A representative of two Coomassie brilliant blue-stained gels is shown for each panel.

To remove the contaminant protein (band E) present in Li-CRP-II, each fraction collected after ion-exchange chromatography was subjected to SDS-PAGE individually (**Figure 2A**), instead of pooling the fractions (lane 2, **Figure 1D**). As shown in **Figure 2A**, the intensity of band E was directly proportional to the intensity of band C and that the compositions of Li-CRP-II present in each fraction were different from each other. Some fractions had more of subunit C and some had more of subunit D. That was not the case with Li-CRP-I (**Figure 2B**); when individual fractions of Li-CRP-I after ion-exchange chromatography were subjected to SDS-PAGE, the composition of Li-CRP-I in each fraction was the same.

Deglycosylation of Li-CRP followed by SDS-PAGE were performed to determine whether each type of subunit present in Li-CRP-I and Li-CRP-II was glycosylated. As shown in **Figure 2C**, deglycosylation of Li-CRP-I resulted in a single band of molecular weight 23.2 kDa (lanes 1 and 2). Similarly, deglycosylation of Li-CRP-II also resulted in a single band of molecular weight 23.2 kDa (lanes 4 and 5). These data suggest that the difference in the electrophoretic mobility between subunits A and B and between subunits C and D was due to the difference in the extent of glycosylation of each type of subunit. Bands A and B were the products of a single gene and bands C and D were also the products of a single gene.

Native PAGE was also performed to check the purity of Li-CRP preparations. As shown in **Figure 2D**, there was only one band for Li-CRP-I, further indicating that it was a pure preparation of Li-CRP-I. In contrast, two bands were observed in the native PAGE of Li-CRP-II. This was not expected. Likely, the top band seen in Li-CRP-II lane contained ligand-bound Li-CRP-II and the ligand could be the protein seen as band E in SDS-PAGE gels.

The N-terminal sequences of the first ten amino acids of Li-CRP-I and Li-CRP-II were LEEGEITSKV and LEEGEITSKI, respectively. The amino acid sequences of Li-CRP-I and Li-CRP-II were identical for the first nine residues. Sequencing of Li-CRP-II, however, revealed an additional protein with the sequence MLTTKVRFFH of the first 10 residues, probably reflecting the band E in Li-CRP-II.

The preparations of Li-CRP-I and Li-CRP-II purified by affinity chromatography and gel filtration as shown in **Figure 1B** were used in the subsequent experiments involving Li-CRP.

### Li-CRP-II reacts with polyclonal antibodies to Li-CRP-I

**Figure 3:**
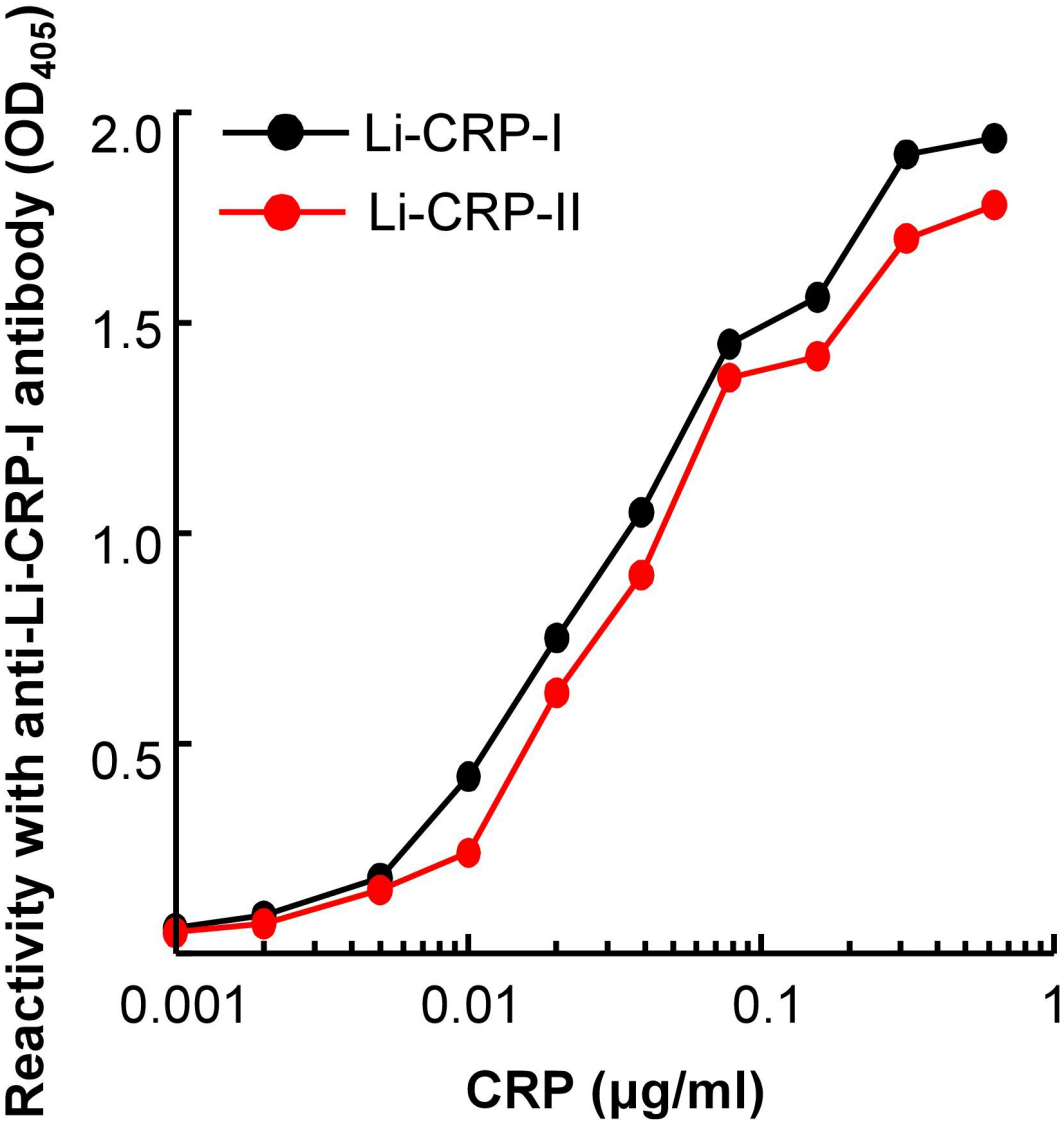
Reactivity of anti-Li-CRP-I antibodies with Li-CRP-II. Microtiter wells were coated with Li-CRP-I and Li-CRP-II. After blocking the unreacted sites in the wells, rabbit polyclonal anti-Li-CRP-I antibody was added to the wells. Bound antibody was detected by HRP-conjugated donkey anti-rabbit IgG. Color was developed and the OD was read at 405 nm. The experiment was performed three times and comparable results were obtained each time. Results of a representative experiment are shown.

Immunological cross-reactivity assays for Li-CRP-I and Li-CRP-II showed that the antibodies to Li-CRP-I reacted with both Li-CRP-I and Li-CRP-II with almost equal affinity (**Figure 3**). These data suggested that anti-Li-CRP-I antibodies could be used to detect both Li-CRP-I and Li-CRP-II. These results were not surprising considering the similarity in the amino acid sequences of Li-CRP-I and Li-CRP-II.

### Li-CRP binds to PCh but not as avidly as human CRP does

**Figure 4:**
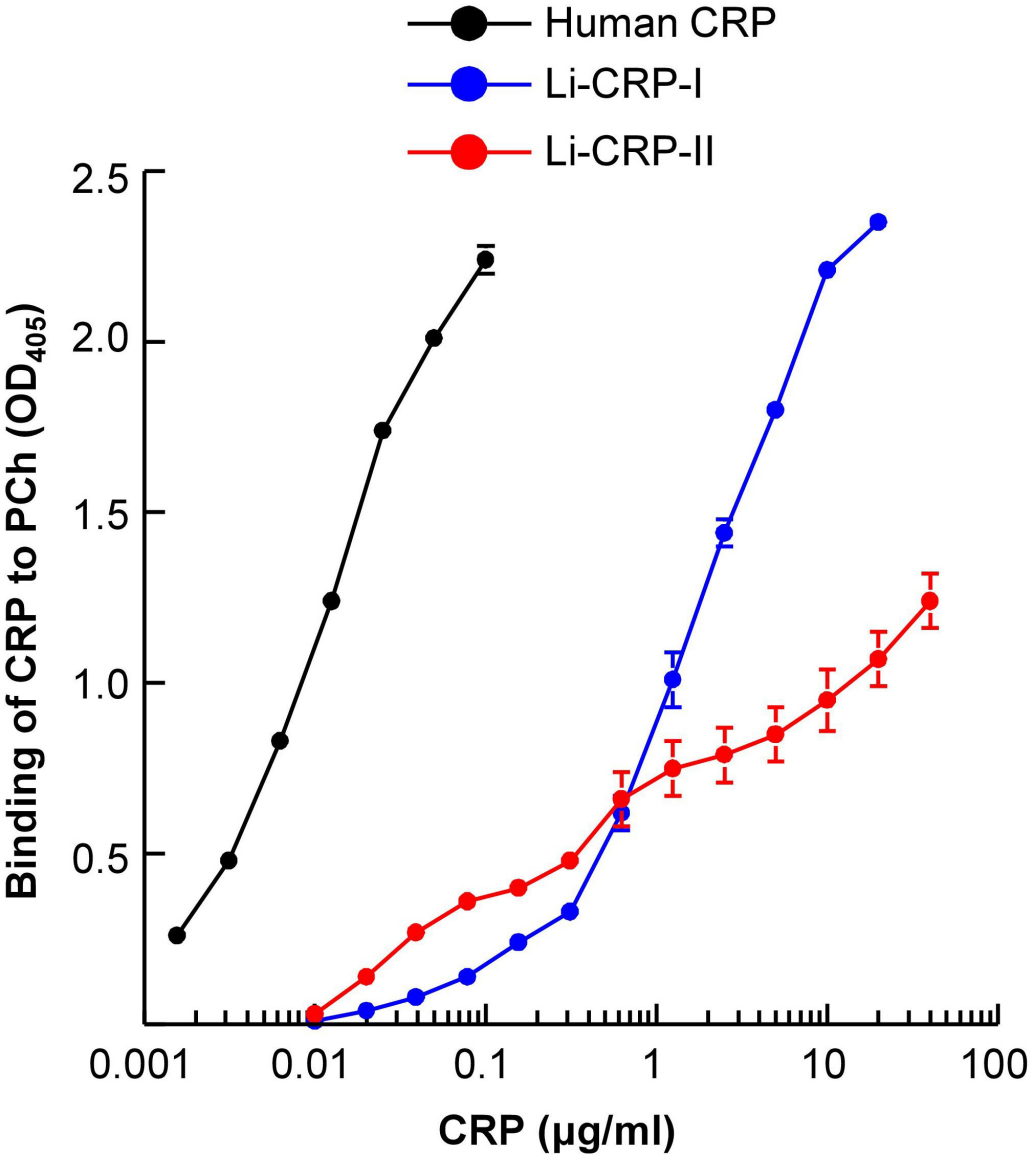
PCh-binding activity of Li-CRP-I and Li-CRP-II. Microtiter wells were coated with PnC. After blocking the unreacted sites in the wells, human CRP and Li-CRP diluted in TBS-Ca were added to the wells. Bound human CRP was detected by using rabbit anti-human CRP antibody and bound Li-CRP was detected by using anti-Li-CRP-I antibody. HRP-conjugated donkey anti-rabbit IgG was used as the secondary antibody. Color was developed and the OD was read at 405 nm. Data shown are mean ± SEM of three experiments.

We next evaluated whether Li-CRP-II which was purified by PEt-affinity chromatography bound to PCh also and, if so, then whether the PCh-binding ability of Li-CRP-II was different from that of Li-CRP-I and human CRP. As shown in **Figure 4**, both Li-CRP-I and Li-CRP-II and human CRP bound to PCh in a CRP concentration-dependent manner. However, the binding of Li-CRP-I and Li-CRP-II to PCh was not comparable to each other and was different from human CRP. For equivalent binding (OD at 405 nm equal to 1) of Li-CRP-I, Li-CRP-II and human CRP to PCh, the required concentrations of Li-CRP-I, Li-CRP-II and human CRP were 1.2 μg/ml, 12 μg/ml and 0.009 μg/ml, respectively. Thus, for equivalent binding of Li-CRP-II and Li-CRP-I to PCh, 10-times more of Li-CRP-II was required compared to Li-CRP-I, indicating that the PCh-binding avidity of Li-CRP-II was 90% less than that of Li-CRP-I. For equivalent binding of Li-CRP-II and human CRP to PCh, ~1300-times more of Li-CRP-II was required compared to human CRP, indicating that the PCh-binding avidity of Li-CRP-II was ~99% less than that of human CRP. Similarly, for equivalent binding of Li-CRP-I and human CRP to PCh, ~130-times more of Li-CRP-I was required compared to human CRP, indicating that the PCh-binding avidity of Li-CRP-I was also ~99% less than that of human CRP.

### Like human SAP, both Li-CRP-I and Li-CRP-II bind to immobilized Aβ at physiological pH

**Figure 5:**
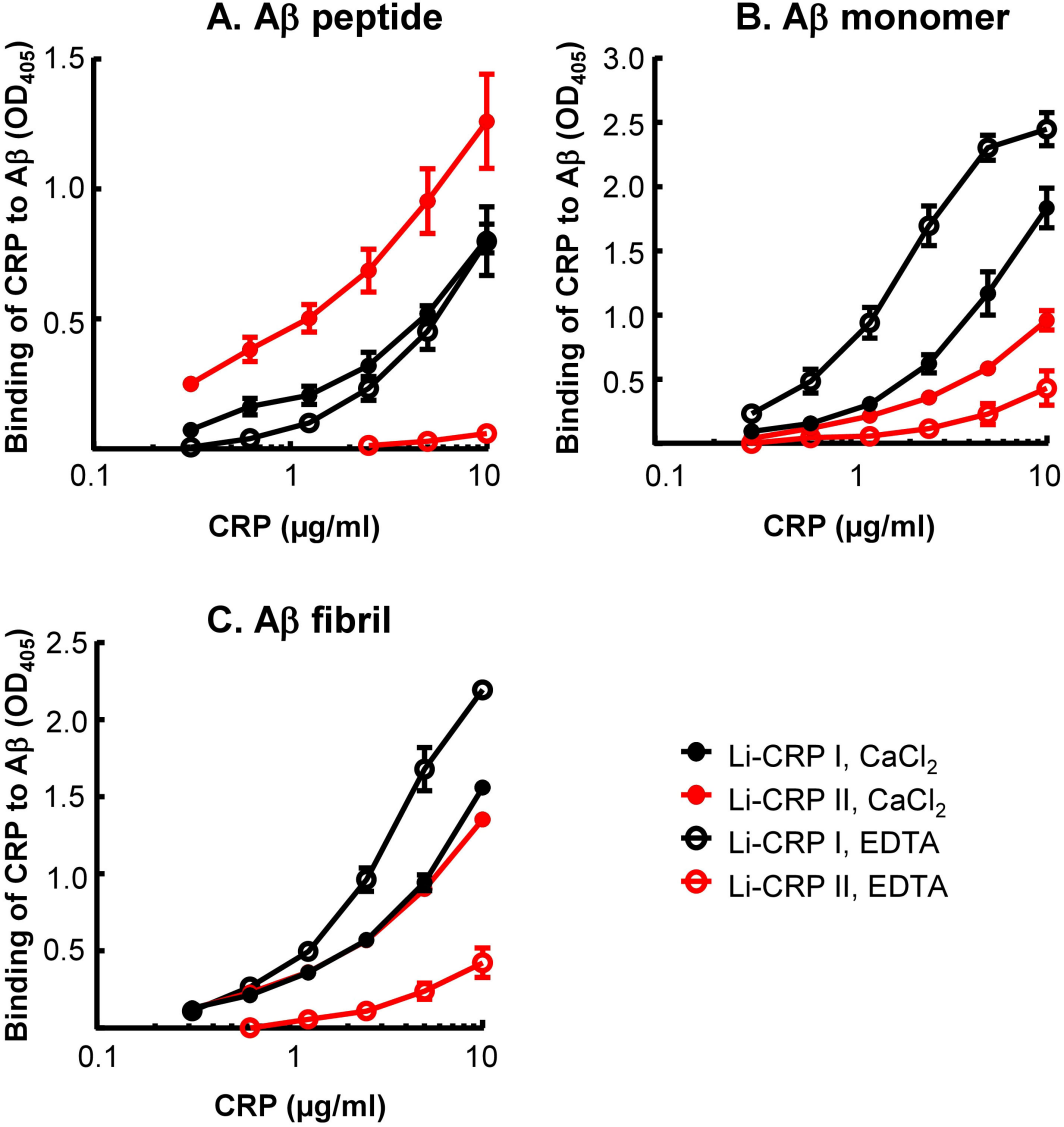
Aβ-binding activity of Li-CRP-I and Li-CRP-II. **(A)** Microtiter wells were coated with Aβ peptides. The unreacted sites in the wells were blocked with gelatin. Li-CRP, diluted in TBS-Ca and TBS-EDTA, was added to the wells. Bound CRP was detected by using anti-Li-CRP-I antibody as the primary antibody and HRP-conjugated donkey anti-rabbit IgG as the secondary antibody. Color was developed and the OD was read at 405 nm. Data shown are mean ± SEM of three experiments. **(B)** As in *A*, except that the microtiter wells were coated with Aβ monomers. **(C)** As in **(A)**, except that the microtiter wells were coated with Aβ fibrils.

Employing solid-phase Aβ-binding assays, the binding of Li-CRP to immobilized Aβ, in the presence and absence of Ca^2+^, was determined. As shown in **Figure 5**, Li-CRP-I bound to immobilized Aβ peptides, monomers and fibrils, at physiological pH, in a CRP concentration-dependent manner. The binding of Li-CRP-I to Aβ did not require Ca^2+^ since the binding also occurred in the absence of Ca^2+^. Similarly, Li-CRP-II also bound to Aβ peptides, monomers and fibrils, at physiological pH, in a CRP concentration-dependent manner. However, in contrast to Li-CRP-I, the binding of Li-CRP-II to Aβ was Ca^2+^-dependent since the binding of Li-CRP-II to Aβ was either absent or drastically reduced in the absence of Ca^2+^. These data suggest that Aβ is a ligand of both native Li-CRP-I and native Li-CRP-II although the undefined Aβ-binding site may be located on different regions in Li-CRP-I and Li-CRP-II.

**Figure 6:**
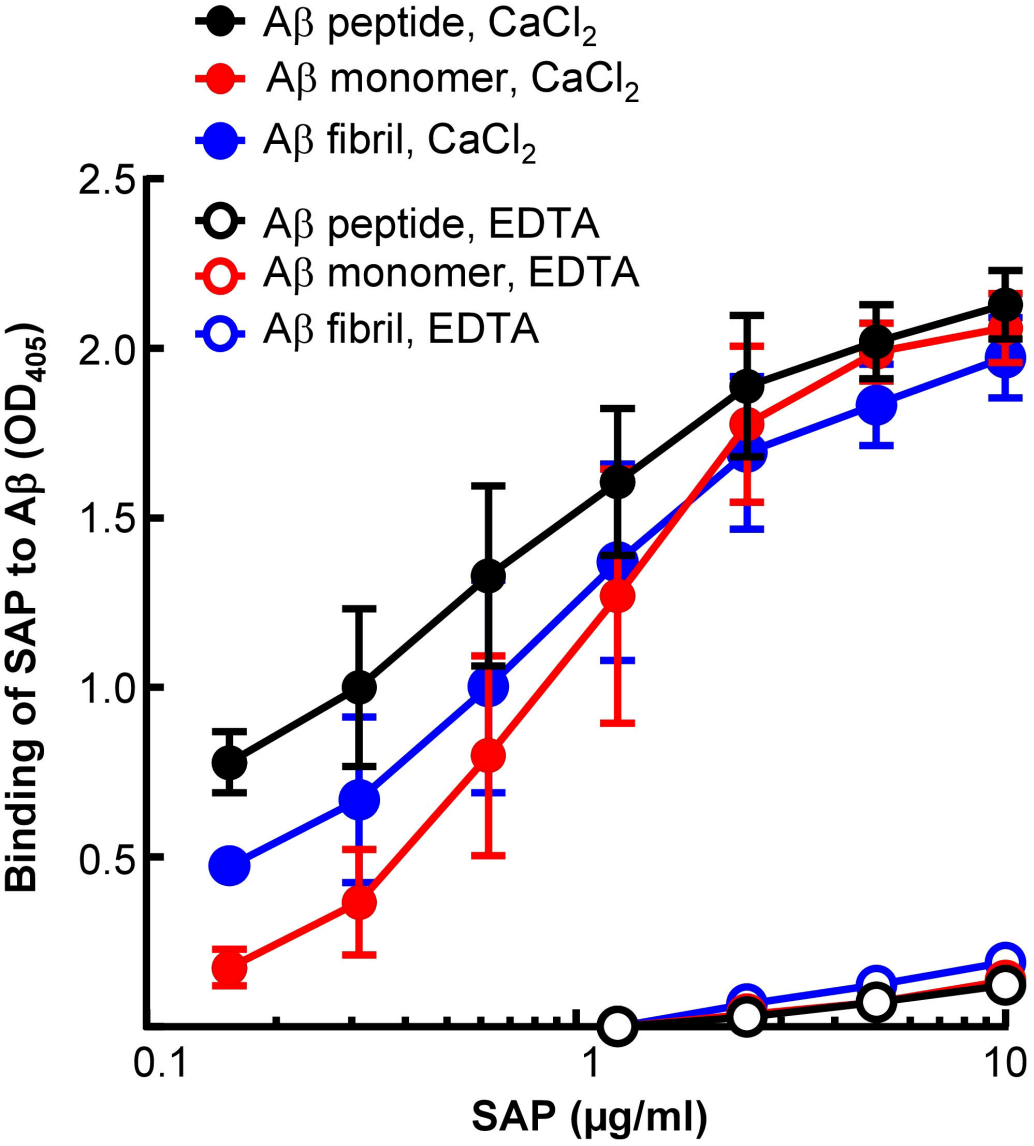
Aβ-binding activity of human SAP. Microtiter wells were coated with Aβ peptides, monomers and fibrils. The unreacted sites in the wells were blocked with gelatin. Human SAP, diluted in TBS-Ca and TBS-EDTA, was added to the wells. Bound SAP was detected by using rabbit polyclonal anti-SAP antibody as the primary antibody and HRP-conjugated donkey anti-rabbit IgG as the secondary antibody. Color was developed and the OD was read at 405 nm. Data shown are mean ± SEM of three experiments.

Next, to compare Li-CRP with human SAP, the binding of human SAP to immobilized Aβ, in the presence and absence of Ca^2+^, was investigated (**Figure 6**). As shown, human SAP bound to immobilized Aβ peptides, monomers and fibrils, at physiological pH, in the presence of Ca^2+^ and in an SAP concentration-dependent manner. However, like Li-CRP-II, human SAP did not bind to Aβ in the absence of Ca^2+^, suggesting that Li-CRP-II and human SAP recognize Aβ in similar environment.

### Both Li-CRP-I and Li-CRP-II prevent fibrillation of Aβ

**Figure 7:**
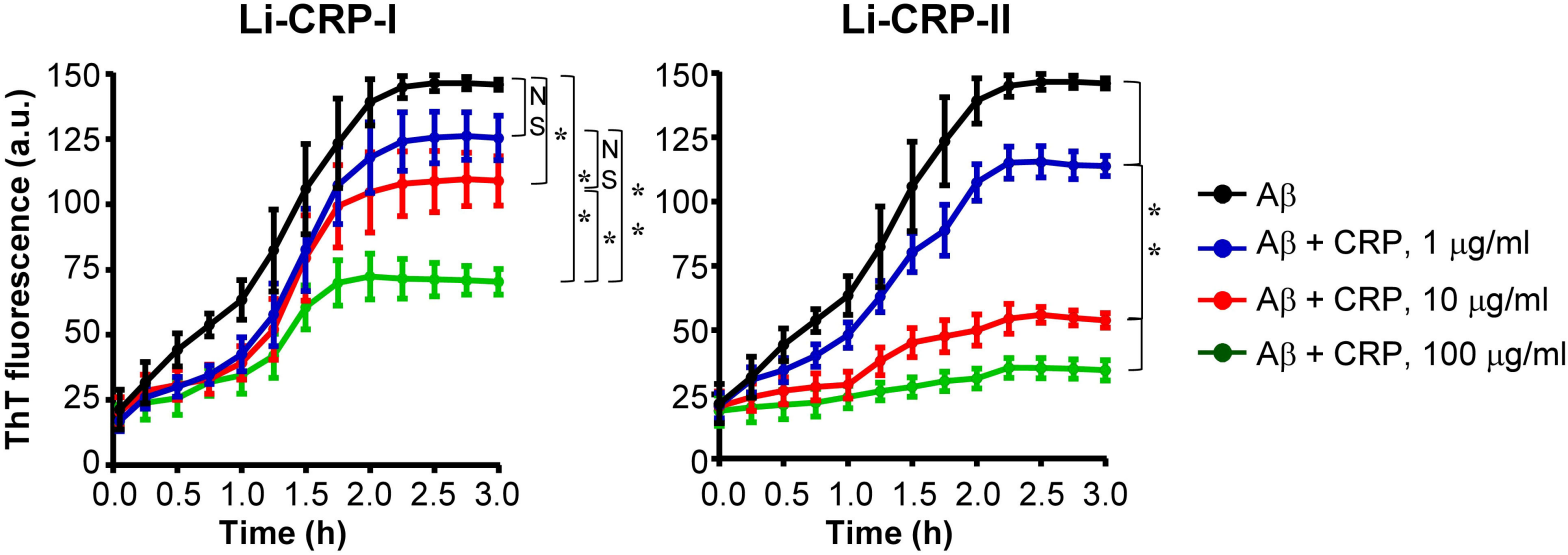
Effects of Li-CRP-I and Li-CRP-II on the formation of Aβ fibrils. The fibrillation of Aβ was measured by ThT fluorescence in the absence (black) or presence of 1 μg/ml (blue), 10 μg/ml (red) and 100 μg/ml (green) of Li-CRP. Li-CRP-I and Aβ monomers were mixed at time zero and added to microtiter wells. After the first measurement at 5 min, the plate was incubated at 37°C with shaking; fluorescence was measured every 15 min. Results are plotted as mean arbitrary units (a.u.) ± SEM of three experiments. For the time period of 5 min to 2 h, *p* values were determined by employing linear regression analysis of the slopes. For the time period of 2.25 h to 3 h, *p* values were determined by taking the mean of all points and employing student unpaired *t* test. NS, not significant (*p*>0.05); * < 0.05; ** <0.008.

Next, we investigated whether Li-CRP capable of binding to immobilized Aβ can also bind to Aβ when both Li-CRP and Aβ are in the fluid phase and inhibit the formation of Aβ fibrils. Aβ monomers were employed in the fibrillation assays (**Figure 7**). In the absence of Li-CRP, the fibrillation of Aβ began within 5 min and continued until ~2 h when further fibrillation stopped (black curves in both panels). Both Li-CRP-I and Li-CRP-II inhibited the fibrillation of Aβ in a CRP concentration-dependent manner. In case of Li-CRP-I, there was no statistically significant difference in fibrillation with or without 1 μg/ml of Li-CRP-I and there was no statistically significant difference in fibrillation with 1 μg/ml and 10 μg/ml of Li-CRP-I. There were statistically significant differences in the fibrillation when Li-CRP-I was used at 10 μg/ml and 100 μg/ml. In case of Li-CRP-II, the inhibition of fibrillation was more efficient; even 1 μg/ml of Li-CRP-II was able to significantly inhibit the fibrillation of Aβ and the inhibition of fibrillation was significantly more at higher concentrations of Li-CRP-II. Thus, both Li-CRP-I and Li-CRP-II bound to Aβ in the fluid phase, inhibited the rate of fibrillation of Aβ over time, and also inhibited the total amount of the fibrils formed by the end of the fibrillation reaction.

## Discussion

In this study, we tested the hypothesis that the recently reported function of human CRP to bind to Aβ peptides and prevent their fibrillation (21) has been evolutionarily conserved. Two different CRP species, termed as Li-CRP-I and Li-CRP-II, were isolated from hemolymph of *L. polyphemus* and investigated for their anti-amyloidogenic activity. Our major findings were: 1. A variety of Li-CRP molecules of different subunit compositions are present in *Limulus* hemolymph. 2. Both Li-CRP-I and Li-CRP-II bound to both immobilized and fluid-phase Aβ. 3. The binding of both Li-CRP-I and Li-CRP-II to Aβ did not require acidic pH or any structural alteration of the Li-CRP dodecamers. 4. Both Li-CRP-I and Li-CRP-II prevented the formation of Aβ fibrils. These findings indicate that Li-CRP is an anti-amyloidogenic protein and raise the possibility that the presence of various Li-CRP species in hemolymph facilitates the recognition of a range of proteins with differing amyloidogenicity.

Before purifying Li-CRP by employing affinity chromatography, the SAP-like protein was removed by passing the hemolymph through a Sepharose column. Also, purified Li-CRP-I and Li-CRP-II did not bind to fetuin when passed through a fetuin-sepharose column (sialic acid affinity chromatography) indicating that both Li-CRP preparations did not contain limulin either (data not shown). The results of the N-terminal sequencing of Li-CRP-I and Li-CRP-II verified the purity of Li-CRP preparations. Both Li-CRP-I and Li-CRP-II were dodecamers with a molecular weight of ~300 kDa consisting of two types of glycosylated subunits, six copies of each, with the same N-terminal amino acid residues as reported previously (33–36). The results of the deglycosylation experiments, combined with the amino acid sequencing data, indicated that the two types of subunits of Li-CRP-I were differentially glycosylated forms of a single gene product. Similarly, the two types of subunits of Li-CRP-II were also differentially glycosylated products of another homologous gene. It is not clear whether the PCh-binding and PEt-binding activities of Li-CRP-I and Li-CRP-II were due to the differences in the extent of glycosylation or due to being the products of homologous genes.

The data obtained from the PCh-binding assays suggested that Li-CRP-II, which was purified by PEt-affinity chromatography, bound to PCh also, although purified Li-CRP-II did not bind to PCh on the PCh-Sepharose column (data not shown). Similarly, Li-CRP-I, which was purified by PCh-affinity chromatography, either did not bind or bound poorly to PEt on the PEt-Sepharose column (data not shown). The PEt-binding activity of Li-CRP-I could not be assessed by using immobilized biotinylated PEt as the PEt-containing ligand (18) since Li-CRP bound to biotin itself. It is possible that Li-CRP-II preparations contained some Li-CRP-I and that Li-CRP-I preparations contained some Li-CRP-II.

Our data indicate that the anti-amyloidogenic function of CRP has been conserved from arthropods to humans. The difference between Li-CRP and human CRP in exerting their anti-amyloidogenic function is that Li-CRP does not require any change in its dodecameric structure while human CRP requires an inflammatory milieu that can change its pentameric structure (8). In this regard, Li-CRP, specifically Li-CRP-II, is more like human SAP. Human SAP also bound to Aβ peptides and fibrils without any structural alteration of SAP and needed Ca^2+^ (23, 51–53). The binding of SAP to Aβ is known to prevent fibrillation and the binding of SAP to the fibrils of Aβ prevents further fibrillation (51–55). The findings that both, Li-CRP which is a dodecamer and human CRP which is a pentamer, bind to Aβ suggest that an Aβ-binding site is located on each subunit. This is reasonable to assume since the Aβ and Aβ-like structures can be accommodated by a single CRP subunit.

To determine the role of the carbohydrate component of Li-CRP in binding to their ligands, Li-CRP was deglycosylated by employing two methods: chemical deglycosylation (**Figure 2C**) and enzymatic deglycosylation (data not shown). Enzymatic deglycosylation was performed under both denaturing and non-denaturing conditions using the Protein Deglycosylation Mix II kit (New England Biolabs, P6044). Both deglycosylation procedures resulted in the denaturation of the protein. Therefore, the role of the carbohydrate moieties present on Li-CRP-I and Li-CRP-II in binding to their ligands could not be evaluated in functional assays.

The data obtained from the biochemical analyses of Li-CRP indicated that Li-CRP-II existed in both free and ligand-bound form in hemolymph. This interpretation is based on the finding that the intensity of the contaminant band E in the gels, possibly a Li-CRP-II ligand, was proportional to the intensity of one of the two Li-CRP-II bands. Also, the protein seen as band E could not be removed by gel filtration and ion-exchange chromatography. The presence of two bands for Li-CRP-II in the native PAGE gels supported the presence of ligand-bound Li-CRP-II in hemolymph. If the protein present in band E is a ligand of Li-CRP-II and could be co-purified with Li-CRP-II by PEt-affinity chromatography, then the data suggest that the PEt-binding site in liganded Li-CRP-II was vacant. Combined data suggest that there are two functional ligand-binding sites on Li-CRP, one site for binding to PEt and the other site for covalent binding to a protein ligand that has amyloid-like structures.

Previously, Li-CRP has been shown to protect against xenobiotic insults and to chelate the heavy metals mercury and cadmium, and hence playing a role in detoxification of heavy metals (56). Li-CRP has also been shown to exhibit Ca^2+^-independent binding to membranes mimicking the outer membrane of gram-negative bacteria and then create pores in the lipid bilayer (57). In addition, Li-CRP also bound to any protein immobilized on microtiter wells which could be due to the exposure of amyloid-like structures on the immobilized proteins (58). We report here that Li-CRP exerts anti-amyloidogenic function. Thus, Li-CRP is a polyfunctional protein. Li-CRP is one of the most abundant constitutively expressed proteins in hemolymph (28, 33–36). The average concentration of Li-CRP in hemolymph is ~2.0 mg/ml. The presence of high concentration of Li-CRP in hemolymph at all times suggests an important function for the protein and a function that is needed at all times. This can be attributed to the fact that these animals are constantly exposed to harsh environments which can induce the formation of amyloidogenic proteins in hemolymph. It is likely that constitutive expression of Li-CRP helps protecting the animals from microbial pathogens and pathogenic proteins by utilizing its PCh-binding and Aβ-binding properties, respectively.

Purified Li-CRP-II was a mixture of dodecamers composed of different types of subunits and hexamers, as suggested by the data obtained from ion-exchange chromatography. If the function of Li-CRP depended upon their glycosylation (35–37), then the random assembly of various types of subunits and hexamers into dodecamers will generate a repertoire of native Li-CRP species where the functional efficiency of each dodecamer will be different. At least nine different Li-CRP species were found to be present in hemolymph in an earlier study (36). Our findings raise the possibility that the presence of various Li-CRP species in hemolymph facilitates the recognition of a range of proteins with differing amyloidogenicity. In a mouse model of amyloidosis, it has been shown previously that injected human SAP was deposited in amyloidotic mice while Li-CRP was not (59). It is possible that Li-CRP was not deposited on amyloids in mice significantly since Li-CRP might have been the mixture of a variety of dodecamers, each being specific for a particular Aβ. Although the cross-reactivity against human CRP and rabbit CRP is weak (28, 39), Li-CRP exhibits immunological cross-reactivity against snail CRP (31), suggesting that CRP performs anti-amyloidogenic functions in invertebrates in general. A mechanism for the generation of many structurally-altered variants of human CRP pentamers has also been reported: both, a wide range of acidic pH and the treatment of CRP with H_2_O_2_ have all been shown to generate CRP pentamers capable of binding to Aβ (8, 9, 18). It is unlikely that the structures of acidic pH-modified CRP molecules and H_2_O_2_-modified CRP (8, 18) will have identical structures.

We conclude that the anti-amyloidogenic function of CRP through the recognition of Aβ is an ancient function of CRP. Our findings that the PCh-binding avidity of Li-CRP was more than 90% lower than that of human CRP suggest that the anti-amyloidogenic function of CRP is its most basic function. In invertebrates, the Aβ-binding function of CRP can protect the host against toxicity caused by amyloidogenic and pathogenic proteins (60). It has been shown previously that the blocking a region in Aβ blocks Aβ toxicity (61). In humans, where CRP is an acute phase protein produced during inflammatory states (62), this property of CRP can protect the host against inflammatory diseases involving malfunctioning proteins such as atherosclerosis in which LDL is ectopically deposited in the arteries, in pneumococcal infection where complement inhibitor factor H is deposited on pneumococci and in inflammatory arthritis in which immune complexes are formed, assuming that these proteins expose Aβ-like structures after deposition at places where they are not supposed to be (63–65).

## Data Availability

The original contributions presented in the study are included in the article/supplementary material. Further inquiries can be directed to the corresponding authors.
